# A predictive model for social participation of middle-aged and older adult stroke survivors: the China Health and Retirement Longitudinal Study

**DOI:** 10.3389/fpubh.2023.1271294

**Published:** 2024-01-12

**Authors:** Yan Liu, Tian Li, Linlin Ding, ZhongXiang Cai, Shuke Nie

**Affiliations:** ^1^Department of Nursing, Renmin Hospital of Wuhan University, Wuhan, China; ^2^Department of Coronary Heart Disease, Renmin Hospital of Wuhan University, Wuhan, China; ^3^School of Nursing, Hubei University of Chinese Medicine, Wuhan, China; ^4^Department of Neurology, Renmin Hospital of Wuhan University, Wuhan, China

**Keywords:** stroke, middle-aged and older adults, social participation, prediction model, nomogram

## Abstract

**Objective:**

This study aims to develop and validate a prediction model for evaluating the social participation in the community middle-aged and older adult stroke survivors.

**Methods:**

The predictive model is based on data from the China Health and Retirement Longitudinal Study (CHARLS), which focused on individuals aged 45 years or older. The study utilized subjects from the CHARLS 2015 and 2018 wave, eighteen factors including socio-demographic variables, behavioral and health status, mental health parameters, were analyzed in this study. To ensure the reliability of the model, the study cohort was randomly split into a training set (70%) and a validation set (30%). The Least Absolute Shrinkage and Selection Operator (LASSO) regression analysis was used to identify the most effective predictors of the model through a 10-fold cross-validation. The logistic regression model was employed to investigate the factors associated with social participation in stroke patients. A nomogram was constructed to develop a prediction model. Calibration curves were used to assess the accuracy of the nomogram model. The model’s performance was evaluated using the area under the curve (AUC) and decision curve analysis (DCA).

**Result:**

A total of 1,239 subjects with stroke from the CHARLS database collected in 2013 and 2015 wave were eligible in the final analysis. Out of these, 539 (43.5%) subjects had social participation. The model considered nineteen factors, the LASSO regression selected eleven factors, including age, gender, residence type, education level, pension, insurance, financial dependence, physical function (PF), self-reported healthy,cognition and satisfaction in the prediction model. These factors were used to construct the nomogram model, which showed a certain extent good concordance and accuracy. The AUC values of training and internal validation sets were 0.669 (95%CI 0.631–0.707) and 0.635 (95% CI 0.573–0.698), respectively. Hosmer–Lemeshow test values were *p* = 0.588 and *p* = 0.563. Calibration curves showed agreement between the nomogram model and actual observations. ROC and DCA indicated that the nomogram had predictive performance.

**Conclusion:**

The nomogram constructed in this study can be used to evaluate the probability of social participation in middle-aged individuals and identify those who may have low social participation after experiencing a stroke.

## 1 Introduction

Stroke is a significant contributor to both mortality and long-term disability, and there is a growing concern about a global stroke epidemic (1). According to data from 2019, stroke ranked as the second leading cause of both death, affecting 6.6 million individuals, and disability, resulting in the loss of 143 million disability-adjusted life years (DALYs) globally (2, 3). According to the Global Burden of Disease Study (GBD) 2017, it was estimated that stroke caused around 2 million deaths in China in 2017, making it the primary cause of years of life lost (4).Stroke is a leading cause of neurological morbidity, particularly among middle-aged and older adult individuals. It frequently leads to lasting neurological deficits, which can have a profound impact on an individual’s quality of life (5). The incidence of stroke is increasing every year, posing a substantial social and economic burden (6). The global population is aging, and this trend is particularly pronounced in China, which has the highest number of older individuals worldwide (7). As a result, China is confronted with substantial challenges in delivering care and addressing the needs of its aging population, particularly those who have suffered from strokes (8). These stroke survivors often face significant obstacles in terms of their social participation due to physical decline, in addition to the mental and spiritual burdens they carry.

There is currently no consensus on the exact definition of social participation (9, 10). However, it is widely recognized as a crucial element in the recovery process of individuals who have experienced a stroke. The International Classification of Functioning, Disability, and Health (ICF) model emphasizes the importance of social participation in restoring functionality (11). A systematic review and network meta-analysis have highlighted the significance of survivors of stroke engaging in meaningful social activities (12). There are studies indicating that social participation is significantly associated with individual-level factors, such as socio-demographic factors, ADL, physical function, depression, cognition and satisfaction.in participating in activities (13–16). On one hand, the older adult can utilize their own strengths and abilities to engage in work or social activities, which not only provides physical exercise but also contributes to society. On the other hand, participating in social interaction activities can enhance the older adult’s mood, helping them overcome negative emotions and maintaining their overall well-being (17). Insufficient social participation in stroke survivors is closely associated with their emotional perception disorders, which may negatively impact their quality of life.Research indicates that stroke patients often face significant challenges in emotional perception. Additionally, these emotional perception issues experienced by stroke patients are strongly linked to social participation and satisfaction with psychological well-being. It has been observed that poor emotional perception is associated with reduced social participation and a lower quality of life. However, it is important to note that this relationship between emotional perception and social participation is not solely due to general cognitive impairment following a stroke (18).

While there is a growing body of research focusing on social participation in stroke, there is currently a dearth of tools available for predicting social participation in stroke patients. This study aims to explore the social participation of middle-aged and older adult individuals and identify the factors that influence their participation. By developing a prediction model, this research can assist social institutions, researchers, and family caregivers in predicting and promoting social participation among this population.

## 2 Methods

### 2.1 Study participants

The China Health and Retirement Longitudinal Study (CHARLS) is a nationally representative longitudinal survey of persons in China 45 years of age or older and their spouses, which was publicly available at http://charls.pku.edu.cn (19).we selected eligible participants in CHARLS 2015 and 2018 to analysis in this study. The cohort was conformed to the Declaration of Helsinki and approved by the Peking University Institutional Review Board (IRB00001052-11015, IRB00001052-13074).

Inclusion criteria were as follows: (1) age ≥ 45 years; (2) Answer the questions about social participation; (3) Complete survey of basic information (gender, age, residence type, education level, marital status, work and retirement); (4) physical function(PF), activity of daily living (ADL), instrumental activity of daily living (IADL), depression, sleep, self-reported health, life satisfaction and pension insurance were investigated; (5) Sample missing value ≤20%.

**Figure 1:**
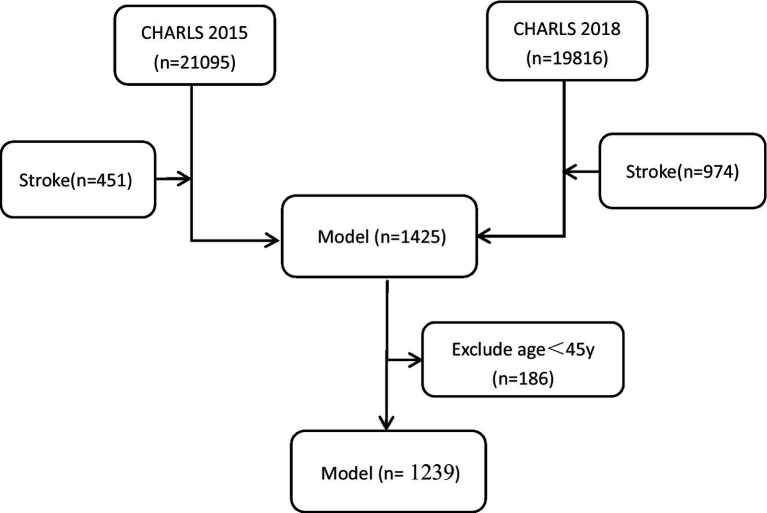
Flowchart for population selection from the CHARLS.

The study data were strictly selected as the criteria above. The data included as the Figure 1, the participants were reported stroke in the CHARLS 2015 wave (n = 451) and 2018 (n = 974) wave included. 1,425 participants selected,the age<45y (n = 186) were exclude. Finally,1,239 eligible participates were included in the prediction model.

### 2.2 Assessment of social participation

The CHARLS survey asked participants to indicate which social activities they engaged in during the previous month. These activities included: (1) interacting with friends;(2) playing mahjong, chess, cards, or participating in table games at a community club; (3) providing unpaid help to family, friends, or neighbors who do not live with them; (4) attending a sport, social, or other type of club, (5) participating in a community-related organization;(6) engaging in voluntary or charity work;(7) caring for a sick or disabled adult who does not live with them;(8) attending an educational or training course;(9) engaging in stock investment;(10) using the Internet; (11) others. For participants who were involved in the activities, the survey also asked about the frequency of their participation. The options provided were: 1 = Almost Daily, 2 = Almost Every Week, 3 = Not Regularly. We categorized individuals who engaged in more than one social activity with a participation frequency of ‘Almost Daily’ or ‘Almost Every Week’ as having social participation (coded as ‘1’), while those who did not meet these criteria were considered to have no social participation (coded as ‘0’) (20, 21).

### 2.3 Predictors

#### 2.3.1 Demographic characteristics

Demographic characteristics included age, gender(1 = male,2 = female), marital status (1 = married and living with spouse/ Cohabitated,2 = separated/divorced/never married/ Widowed), type of residence (1 = urban,2 = rural), education (1 = Elementary school or below,2 = Middle school,3 = High school or above), the pension, insurance, income (1 = yes,2 = no),work and retirement (1 = Currently Not Working,/Never Worked, 2 = Working).

#### 2.3.2 Health status and behavior

Health status and behavior included physical function(PF), activity of daily living (ADL),instrumental activity of daily living (IADL), sleep duration, body pain. Physical functioning (PF) is assessed through various activities such as jogging, walking, climbing stairs, bending down, stretching arms, and lifting heavy objects. Participants rate their level of difficulty in performing these activities on a scale of 1 to 4, with 1 indicating no difficulty, 2 indicating some difficulty but still manageable, 3 indicating difficulty requiring assistance, and 4 indicating inability to perform the activity. The scores of all options are added together to determine the level of PF dysfunction. A higher score indicates a greater impairment in physical functioning. The ADL and IADL evaluating the status of basic and instrumental activities of daily living, respectively, were assessed as continuous numerical variable. In the CHARLS data the ADL assessed by six aspects including dressing, bathing, eating, toilet, transfer and controlling urination and defecation, while the IADL assessed by five aspects including cooking, shopping, taking Medications, managing Money (8).Each question had four possible answers in CHARLS. We have unified code as follows:4 = do not have any difficulty;3 = have difficulty but Can Still Do It;2 = have difficulty and need help;1 = cannot do it. Each option is added to produce a score, with higher scores indicating the better daily living activities. Sleep duration at night is a continuous numerical variable, and the pain was assessed five levels (1 = none, 2 = a little, 3 = somewhat; 4 = quite a bit; 5 = very).

#### 2.3.3 Mental health parameters

The study included mental health parameters such as depression, satisfaction, cognition, self-reported health, and financial dependence. Depressive symptoms were evaluated using the widely used 10-item Center for Epidemiological Studies Depression Scale (CESD-10). This scale assesses both depressed mood and positive affect and consists of ten items. Scores on the CESD-10 range from 0 to 30, with higher scores indicating more severe depressive symptoms (22). Satisfaction is measured through five aspects: self-comment on life-as-a-whole, health, marriage, children, and air quality (23).The option for each aspect is scored in reverse order, with a higher score indicating a higher degree of satisfaction. The total score represents overall satisfaction. The self-reported healthy item is a variable that represents grades, with the answer options being 1 = Very Good, 2 = Good, 3 = Fair, 4 = Poor, and 5 = Very poor. The evaluation of financial dependence involves coding dependent children as “1,” and dependence on pension/saving/insurance/other as “2.” The cognitive used the Minimum Mental State Examination scale (MMSE) including memory, executive function, and orientation domains. Both the validity and the reliability of these scale have been well documented (24).To assess overall cognitive function, z-scores were generated for each cohort using a two-step process. In step 1, the range test scores were normalized to the baseline and domain z-scores were calculated. This involved subtracting each range test score from the mean and dividing it by the standard deviation (SD) of the baseline scores. In step 2, the mean of the three domains was renormalized to the baseline and individual global z-scores were calculated for each wave. This method of generating cognitive z-scores is widely recognized and accepted in the field (25–27).

### 2.4 Statistical analysis

Statistical analysis and drawing were performed using R software. Missing data values were multiple interpolated using Random Forest algorithm. Categorical variables were described using frequency and percentage, while continuous variables were described using mean (M) ± standard deviation (SD) or median (P25–75). Comparisons between groups were performed using t-test, chi-square test and non-parametric test. Data were randomly divided into training (n = 867) and validation (n = 372) sets, according to a ratio of 7:3.

A nomogram was utilized to visually represent the likelihood of social participation in individuals with stroke. Additionally, the LASSO regression analysis was employed to develop and validate the model (28, 29). Initially, the training set data underwent LASSO regression to identify predictors of social participation in stroke patients. Subsequently, tenfold cross-validation was conducted to determine the appropriate tuning parameters (λ) for LASSO regression analysis, and the most significant features were identified using the LASSO algorithm. Finally, the selected predictors were included in a multifactor logistic regression analysis, and a nomogram was constructed (30). The discrimination ability of the model was determined by using the area under the receiver operating characteristic (ROC) curve (AUC). The degree of agreement between predicted probabilities and observed outcomes was determined by using calibration curves. Clinical validity was assessed using decision curve analysis (DCA).

## 3 Results

### 3.1 Participant characteristics

A total of 1,239 individuals with stroke were included in this study. The demographic and clinical characteristics of the participants can be found in Table 1. The ratio of social participation in the study was 43.5% (539/1239). Several factors, including age, residence, education, pension, income, financial dependence, PF, ADL, self-reported healthy, cognition, satisfaction differed significantly (*p* < 0.05) between patients with and without social participation. Univariate analysis was conducted on both the development set and internal validation set (refer to Additional file).

**Table 1:** Baseline characteristics of the study population.

	Non-social participation *(N = 700)*	social participation (*N = 539*)	*p*
Age	59.7 (8.46)	57.6 (8.10)	<0.001
Gender			0.059
Male	373(53.3)	258(47.9)	
Female	327(46.7)	281(52.1)	
Marital			0.843
married and living with spouse/ Cohabitated	516(73.7)	400(74.2)	
separated/divorced/never married/ Widowed	184(26.3)	139(25.8)	
Residence			<0.001
Town	120(17.1)	142(26.3)	
Rural	580(82.9)	397(73.7)	
Education			<0.001
Elementary school or below	527(75.3)	20(2.9)	
Secondary school	153(21.9)	360(66.8)	
College and above	20(2.9)	143(26.5)	
Work and retirement			0.008
Currently Not Working /Never Worked	490(70.0)	210(30)	
Working	210(30)	335(62.2)	
Pension			<0.001
Yes	125 (17.9%)	155 (28.8%)	
No	575 (82.1%)	384 (71.2%)	
Insurance			0.153
Yes	683(97.6)	532(98.7)	Yes
No	17(2.4)	7(1.3)	No
Income			0.024
Yes	52 (7.43%)	60 (11.1%)	
No	648 (92.6%)	479 (88.9%)	
Financial dependence			0.012
Children	402(57.4)	271(50.3)	
pension/saving/insurance/other	298(42.6)	268(49.7)	
PF	18.2 (2.38)	16.8 (2.16)	<0.001
ADL	6.84 (2.65)	5.91 (2.70)	<0.001
IADL	5.65 (1.06)	5.61 (0.99)	0.525
Sleep duration	6.04 (2.59)	6.01 (2.17)	0.847
Pain			0.377
None	198 (28.3%)	169 (31.4%)	
A little	184 (26.3%)	147 (27.3%)	
Somewhat	98 (14.0%)	72 (13.4%)	
Quite a Bit	102 (14.6%)	81 (15.0%)	
Very	118 (16.9%)	70 (13.0%)	
Self-reported healthy:			<0.001
Very Good	17 (2.43%)	22 (4.08%)	
Good	28 (4.00%)	32 (5.94%)	
Fair	209 (29.9%)	229 (42.5%)	
Poor	317 (45.3%)	191 (35.4%)	
Very poor	129 (18.4%)	65 (12.1%)	
Depression	8.98 (7.88)	8.73 (6.94)	0.566
Cognition	−0.91 (1.87)	−0.25 (1.93)	<0.001
Satisfaction	12.0 (6.79)	13.9 (5.39)	<0.001

PF, physical function; ADL, activity of daily living; IADL, instrumental activity of daily living.

### 3.2 Predictive model development

**Figure 2:**
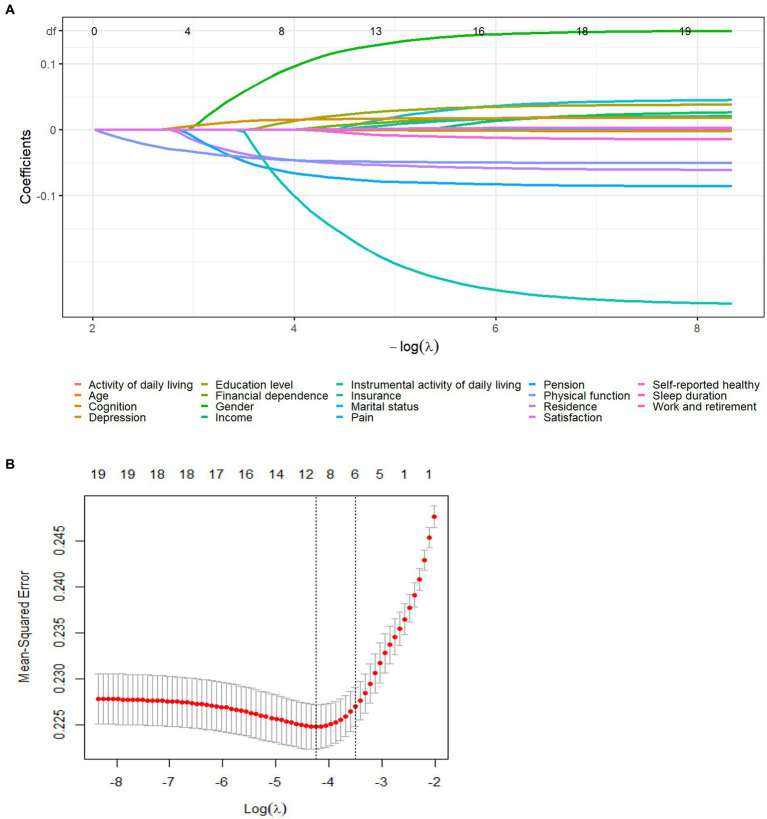
Demographic and clinical feature selection using the LASSO regression model. **(A)** According to the logarithmic (lambda) sequence, a coefficient profile was generated, and non-zero coefficients were produced by the optimal lambda. **(B)** The optimal parameter (lambda) in the LASSO model was selected via tenfold cross-validation using minimum criteria. The partial likelihood deviation (binomial deviation) curve relative to log (lambda) was plotted. A virtual vertical line at the optimal value was drawn using one SE of minimum criterion (the 1-SE criterion). Choose the minimum Lambda = 0.0215, ie log(Lambda) = −3.84.

The participants were randomly assigned to the training and validation sets in a 7:3 ratio. LASSO regression analysis was employed to identify the best predictors for the model, using a 10-fold cross-validation. Non-zero coefficients were selected as potential predictors of social participation (Figures 2A,B). These potential factors were then incorporated into the logistic regression model. The results of the logistic regression can be found in Table 2. The variance inflation factor (VIF) test was conducted, and all variables had VIF values below 4. The model fit is fine without covariance. The variables included age, gender, residence, education, pension, insurance, physical function, financial dependence, self-reported healthy, cognition, and satisfaction as predictors.

**Table 2:** The prediction model with multivariate logistic regression.

	OR	95%CI	*p*
Intercept	22.870	[0.949,0.949]	0.004
Age	0.994	[−0.026,0.013]	0.520
Gender			
Male	Reference		
Female	0.494	[−1.023, −0.392]	<0.001
Residential			
Town	Reference		
Rural	1.328	[−0.124,0.691]	0.173
Education			
Elementary school or below	Reference		
Secondary school	1.530	[−0.345,1.228]	0.287
College and above	1.059	[−0.321,0.433]	0.767
Pension			
Yes	Reference		
No	1.541	[−0.030, 0.895]	0.067
Insurance			
Yes	Reference		
No	3.697	[0.030, 2.881]	0.064
Financial dependence	1.044	[−0.292, 0.377]	0.800
Children	Reference		
Pension/saving/other	0.800	[−0.292,0.377]	0.800
Physical function	0.790	[−0.309, −0.163]	<0.001
Self-reported healthy			
Very Good	Reference		
Good	1.490	[−0.284,1.098]	1.490
Fair	0.855	[−0.497,0.184]	0.855
Poor	1.310	[−0.539,1.095]	1.310
Very poor	1.160	[−0.328,0.623]	1.160
Cognition	0.006	[0.006,0.178]	0.036
Satisfaction	−0.019	[−0.019,0.034]	0.563

**Figure 3:**
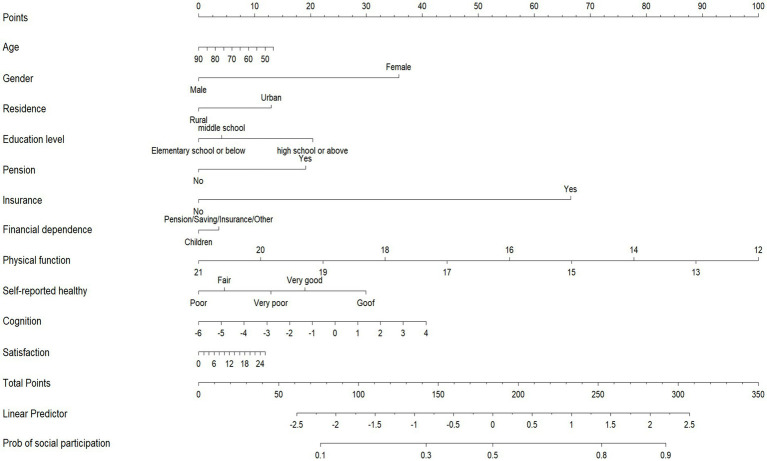
Nomogram.

A nomogram was used to present the predictive model, allowing for quantitative probably prediction of social participation in patients with strokes (Figure 3). The nomogram showed that Physical Function (≤12) corresponded to the highest probable score (100 points),followed by having insurance (67 points). When the influence factors of social participation were visualized, the prob. of individual social participation could be predicted. First, each independent influence factor was projected upward to the first line of the scale to get score of each factor, and then the scores of 11 influence factors were added to get the total scores. Second, the prob. of social participation was calculated according to the total scores. Finally get the predicted probability at the bottom of the nomogram based on the total score.The higher the total scores, the higher the prob. of individual social participation.

### 3.3 Predictive model validation

#### 3.3.1 Discrimination

**Figure 4:**
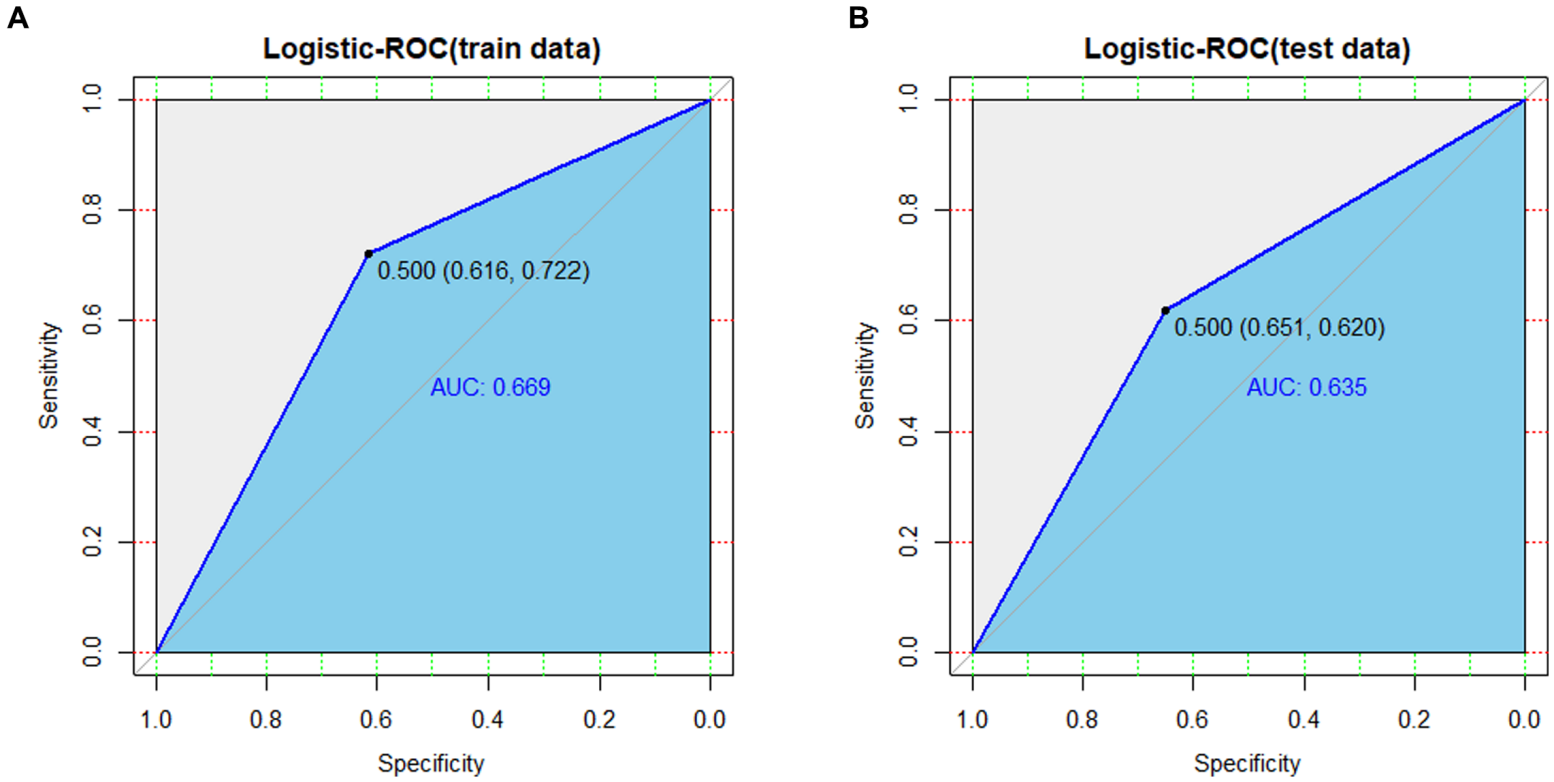
**(A)** ROC curves generated from the training data set. **(B)** ROC curves generated using the validation data set.

AUC values were calculated to assess the discrimination of the predictive model in predicting social participation in stroke patients. The results, shown in Figures 4A,B, indicate that the predictive model achieved an AUC value of 0.669 (95% CI = 0.631–0.707) in the training set, with a specificity of 0.616 and sensitivity of 0.722. In the validation set, the AUC was also 0.635 (95% CI = 0.573–0.698), with a specificity of 0.651 and sensitivity of 0.620. These findings suggest that the nomogram has some discriminatory power and predictive value.

#### 3.3.2 Calibration of the predictive model

**Figure 5:**
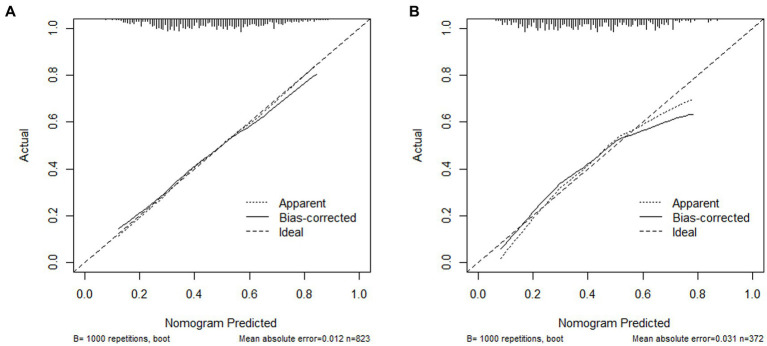
**(A)** Calibration plot for the training data set; **(B)** Calibration plot for the validation data set.

The nomogram was evaluated using a calibration plot and the Hosmer–Lemeshow goodness-of-fit test (*P*>0.05) indicates that the model exhibits a very good degree of fit). The test results showed that the model had a good fit for the training set (X^2^ = 6.5352, df = 8, *p* = 0.5875) and the validation set (X^2^ = 6.7573, df = 8, *p* = 0.563).Calibration plots for the training and validation sets, based on the logistic regression model, are displayed in Figures 5A,B. The calibration curves for the nomogram demonstrate a high level of consistency between the predicted and actual probabilities of frailty in both the training (Figure 5A) and validation (Figure 5B) sets.

#### 3.3.3 Evaluation of clinical validity

**Figure 6:**
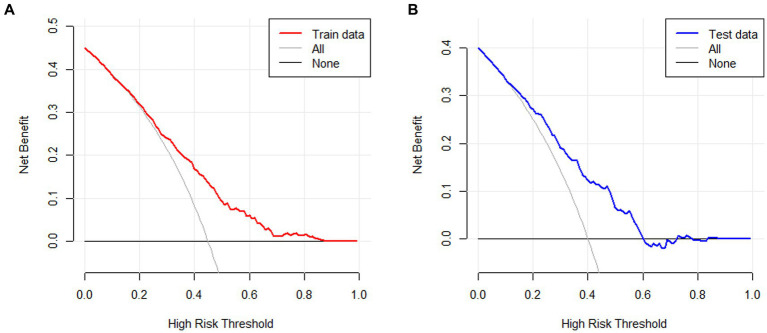
**(A)** DCA curves for the training data set. **(B)** DCA curves for the validation data set.

The clinical validity of the model was evaluated using the DCA method, and the results are shown in Figures 6A,B. From the decision curves, the net benefits of the predictive model for the internal validation set were significantly higher than those of the two extreme cases, indicating that the nomogram model had the superior net benefit and predictive accuracy.

## 4 Discussion

This study established and verified a nomogram model that can predict social participation among middle-aged and older adult Chinese individuals who have suffered a stroke. The findings reveal that 56.5% of stroke survivors did not engage in any social activities. Lower levels of social participation can contribute to feelings of loneliness and social isolation, ultimately hindering post-stroke recovery. Additionally, this can negatively impact the quality of life for individuals who have experienced a stroke and place an increased burden on their families in terms of caregiving responsibilities.Previous studies have demonstrated that increased social participation positively influences the recovery of stroke patients (12, 31–34). Hence, it is essential to identify individuals with limited social participation to implement preventive and intervention measures that promote and enhance social engagement.

The potential for social participation is a complex issue influenced by multiple factors. This study highlights that physical function (PF) is the primary factor impacting the social participation of stroke survivors. The severity of PF damage, as indicated by higher scores, is negatively correlated with social participation. As individuals age, their physical function tends to decline, which is also negatively associated with social participation. This finding aligns with previous research demonstrating a negative correlation between age and PF impairment with social participation (14, 15, 34). Furthermore, our study findings indicate that women exhibit a higher tendency to participate in social activities as compared to men. This aligns with the research conducted by Cai et al. (16) However, it contradicts certain reports suggesting that men are more likely to engage in social activities than women (35, 36). This discrepancy could potentially be attributed to various factors such as biological differences between genders, inconsistencies in the social division of labor, and racial disparities (37). Moreover, individuals residing in urban areas exhibit a greater likelihood of social participation compared to those in rural areas. This disparity could be attributed to factors such as economic conditions, infrastructure, social support, and population density. Interestingly finding, individuals with higher levels of education are more likely to engage in social activities. The results were consistent with recently published finding (15, 16, 21).

Our predictive model revealed a significant association between pension, insurance, financial dependence and social participation in stroke patients. The results indicated that individuals who had pension and insurance were more inclined to actively engage in society after experiencing a stroke, potentially because they felt financially secure. Additionally, we observed that individuals who relied on pension, savings, or insurance rather than their children for financial support tended to exhibit higher levels of sociability. This could be attributed to the psychological burden and enhanced financial stability associated with such arrangements. Moreover, there is a positive correlation between life satisfaction and self-reported health with social participation., indicating that higher levels of self-satisfaction and better self-reported healthy are associated with increased likelihood of social engagement. This finding aligns with previous research conducted by Della Vecchia etc. (38) Cognitive function, comprising orientation, memory, and executive ability, also plays a crucial role. The overall cognitive score is positively correlated with social participation, suggesting that better cognitive function is linked to a higher probability of social engagement. This is consistent with previous studies (36, 39). Furthermore, the study’s results emphasize the importance of cognitive function in middle-aged and older adult stroke patients as a key indicator post-stroke, as it is closely tied to their quality of life and level of social participation.

Social participation is a crucial aspect of stroke rehabilitation and a policy framework for addressing population aging. Factors such as patient demographic characteristics, functional status, and mental conditions play a significant role in determining social participation. While there have been numerous studies in China focusing on enhancing the social participation and integration of post-stroke patients to improve their quality of life and reduce burden, there is a lack of research on the potential influencing factors of social participation and a scarcity of relevant tools for predicting social participation in different groups of stroke survivors. The prediction model developed in this study effectively identifies the factors that affect social participation among high-risk patients and stroke survivors. Our findings indicate that lower physical dysfunction and higher cognitive function are associated with greater social participation after stroke. Although there has been considerable attention on physical functioning and cognitive function after stroke, there is limited knowledge about the relationship between social participation and these factors. Furthermore, our study reveals that women, individuals living alone, and those with higher pensions, social insurance, self-rated health, and satisfaction are more likely to engage in social activities after stroke. These findings emphasize the need to focus on providing support in these areas and addressing the deficiencies in these aspects among stroke survivors.

Our internally verified nomogram model has been found to be a valuable tool for assessing the probability of strokes in patients and their potential for social participation.However, it is important to acknowledge the limitations of the present study. Firstly, the CHARLS database does not provide information on certain potential factors such as community facilities, disease stage classification, and social relationships. This lack of data may have impacted the accuracy of our findings. Secondly, the survey method used in this study was self-report, which is known to be highly subjective. When answering measurement questions, subjects can be influenced by their own subjective consciousness and may intentionally or unintentionally provide selective or socially expected answers, leading to inaccurate measurement results. Factors such as memory bias, social desirability, self-protection, and language expression can also affect the accuracy and reliability of self-reported results. Additionally, the individual’s emotional and psychological state, as well as environmental factors, can introduce errors in the measurement results.Thirdly,it is worth noting that this study was retrospective in nature and the nomogram was developed using data specifically from China. Therefore, caution should be exercised when generalizing these findings, as they may only be applicable to community-dwelling survivors of stroke in China. To enhance the reliability and applicability of our current nomogram model, further verification using data from external cohorts is necessary.

## 5 Conclusion

We developed a predictive model to assess the probability of social participation in middle-aged and older stroke adults. The model considers various factors including age, gender, residence, education level, pension, insurance, financial dependence, physical fitness, self-reported healthy, cognition, and satisfaction. By utilizing the assessment results, early intervention can be implemented in stroke groups to improve social participation.

## Data availability statement

The original contributions presented in the study are included in the article/supplementary material, further inquiries can be directed to the corresponding authors.

## Ethics statement

Written informed consent was obtained from the individual(s) for the publication of any potentially identifiable images or data included in this article.

## Author contributions

YL: Conceptualization, Funding acquisition, Project administration, Supervision, Writing – original draft, Writing – review & editing. TL: Writing – review & editing, Data curation, Investigation, Methodology, Software, Writing – original draft. LD: Writing – review & editing, Conceptualization, Project administration, Supervision. ZC: Conceptualization, Data curation, Methodology, Software, Writing – review & editing. SN: Funding acquisition, Supervision, Writing – review & editing.
